# Supplementary material for: Transcriptome Remodeling and Adaptive Preservation of Muscle Protein Content in Hibernating Black Bears
Source: Ecol Evol. 2025 Jun 26;15(7):e71669. doi: 10.1002/ece3.71669 (PMC12202773; doi:10.1002/ece3.71669)
Supplement: Supplementary file 1 — Table S1. [file ECE3-15-e71669-s001.docx]

**Transcriptome remodeling and adaptive preservation of muscle protein content in hibernating black bears**

Vadim B. Fedorov, Arthur Garreau, Øivind Tøien, Brian M. Barnes, Anna V. Goropashnaya

**Table 1S**. Differentially expressed genes in quadriceps of hibernating black bears as compared to summer active animals. Genes symbols in bold demonstrated expression changes in the same direction in muscle of hibernating brown bears (Jansen et al. 2019).

| Gene Name | Gene Log₂ FC | Gene Fold change | FDR p-value |
| --- | --- | --- | --- |
| OTUD1 | 3.629205 | 12.3737 | 0 |
| BTG2 | 3.420692 | 10.70855 | 1.43E-13 |
| **FOS** | 3.383432 | 10.43553 | 1.23E-07 |
| **ANKRD24** | 3.340664 | 10.13072 | 1.44E-07 |
| NR4A2 | 3.234304 | 9.410711 | 2.19E-06 |
| LOC123775535 | 3.189072 | 9.120242 | 3.76E-12 |
| **EGR1** | 3.031936 | 8.179067 | 1.07E-08 |
| KASH5 | 2.78647 | 6.899393 | 2.83E-05 |
| FAM83H | 2.657078 | 6.307542 | 1.43E-13 |
| EGR3 | 2.646619 | 6.26198 | 0.00267 |
| **FOSB** | 2.608972 | 6.10069 | 0.000182 |
| **SCN3B** | 2.526798 | 5.762911 | 3.68E-07 |
| **ANKRD34A** | 2.473959 | 5.555664 | 0.002068 |
| LOC123788650 | 2.441466 | 5.431936 | 5.86E-06 |
| LOC123795245 | 2.440557 | 5.428513 | 0.000484 |
| FOXH1 | 2.369311 | 5.166942 | 0.000108 |
| ACHE | 2.363485 | 5.146119 | 6.42E-05 |
| LOC123792575 | 2.345772 | 5.083325 | 0.00012 |
| LOC123795656 | 2.339619 | 5.061689 | 0.000916 |
| TPPP2 | 2.320547 | 4.995215 | 5.66E-06 |
| LOC123799362 | 2.293118 | 4.901144 | 0.000382 |
| STC2 | 2.272907 | 4.83296 | 1.38E-05 |
| CCN1 | 2.265868 | 4.809436 | 8.34E-09 |
| **ANGPTL4** | 2.253778 | 4.7693 | 0.000583 |
| LOC123792079 | 2.246745 | 4.746107 | 0.007281 |
| APOLD1 | 2.233088 | 4.701392 | 3.11E-08 |
| **YJEFN3** | 2.202379 | 4.602377 | 0.001309 |
| LOC123803746 | 2.180991 | 4.534649 | 0.00131 |
| LOC123790986 | 2.127127 | 4.368466 | 5.86E-07 |
| LOC123788012 | 2.123724 | 4.358175 | 1.59E-06 |
| ANKRD1 | 2.110254 | 4.317673 | 0.000736 |
| IGF2 | 2.093929 | 4.269092 | 0.002001 |
| LOC123779211 | 2.064159 | 4.181902 | 0.000128 |
| LOC123797260 | 1.944882 | 3.850064 | 8.69E-07 |
| NR4A1 | 1.935591 | 3.825348 | 2.08E-06 |
| LOC123797704 | 1.913365 | 3.766866 | 0.005283 |
| EGR2 | 1.905851 | 3.7473 | 0.043602 |
| LOC123783672 | 1.890644 | 3.708008 | 0.000399 |
| GRM2 | 1.884557 | 3.692396 | 0.005109 |
| **KLF2** | 1.883712 | 3.690232 | 3.61E-08 |
| CHRM4 | 1.872864 | 3.662589 | 0.001896 |
| LOC123795371 | 1.872526 | 3.661731 | 0.024054 |
| **SYCP2** | 1.838931 | 3.577448 | 0.010182 |
| LOC123799834 | 1.815071 | 3.518769 | 0.002045 |
| **SNAI3** | 1.803865 | 3.491542 | 0.000191 |
| PPP1R13L | 1.803365 | 3.490335 | 9.01E-05 |
| PGAP4 | 1.79975 | 3.481598 | 3.68E-07 |
| LOC123798823 | 1.795849 | 3.472198 | 0.005586 |
| KLF4 | 1.789181 | 3.456188 | 4.63E-05 |
| VSIG10L | 1.774767 | 3.421826 | 0.014002 |
| **ANGPT4** | 1.765673 | 3.400325 | 5.94E-08 |
| TNNI3 | 1.76415 | 3.396737 | 0.000139 |
| **DRC1** | 1.74393 | 3.349463 | 0.013926 |
| CALR3 | 1.743467 | 3.348388 | 0.006251 |
| GLTPD2 | 1.740441 | 3.341372 | 0.009663 |
| **UBAP1L** | 1.737818 | 3.335304 | 0.002143 |
| LOC123795871 | 1.726877 | 3.310105 | 0.036695 |
| LOC123794820 | 1.725983 | 3.308053 | 1.72E-06 |
| DUSP1 | 1.717227 | 3.288038 | 1.44E-07 |
| LOC123803736 | 1.715511 | 3.28413 | 0.005794 |
| **SH3YL1** | 1.701044 | 3.251362 | 0.011123 |
| LOC123779349 | 1.700076 | 3.24918 | 0.003569 |
| LOC123794226 | 1.693342 | 3.234049 | 0.011962 |
| LOC123787601 | 1.692003 | 3.23105 | 0.000481 |
| LOC123792964 | 1.690771 | 3.228293 | 0.002045 |
| LOC123801421 | 1.676761 | 3.197093 | 0.010128 |
| **NOG** | 1.67459 | 3.192287 | 0.009898 |
| TAMALIN | 1.66914 | 3.18025 | 0.001962 |
| LOC123793023 | 1.660945 | 3.162237 | 6.49E-05 |
| SEPTIN12 | 1.658447 | 3.156764 | 0.036622 |
| LOC123776668 | 1.645739 | 3.129081 | 0.011322 |
| OTC | 1.635648 | 3.107271 | 0.04442 |
| **GATM** | 1.633387 | 3.102406 | 1.86E-06 |
| KCNQ4 | 1.6271 | 3.088914 | 0.000527 |
| TAS1R1 | 1.6251 | 3.084635 | 0.02124 |
| HABP2 | 1.620519 | 3.074856 | 0.000251 |
| LOC123786631 | 1.619626 | 3.072953 | 0.039956 |
| **GADD45B** | 1.616509 | 3.066321 | 0.000828 |
| LOC123794451 | 1.614469 | 3.06199 | 0.020358 |
| LOC123797794 | 1.607229 | 3.04666 | 0.049055 |
| LOC123776789 | 1.578471 | 2.986532 | 0.034756 |
| LOC123790937 | 1.57665 | 2.982764 | 0.040031 |
| CSRNP1 | 1.56389 | 2.956499 | 0.000286 |
| LOC123777868 | 1.548202 | 2.924525 | 0.010923 |
| EPCAM | 1.539394 | 2.906723 | 0.0166 |
| MAFF | 1.533477 | 2.894827 | 0.005558 |
| **CDK5R1** | 1.530625 | 2.88911 | 0.016624 |
| LOC123779213 | 1.528736 | 2.88533 | 0.014677 |
| **SOX17** | 1.524154 | 2.87618 | 3.41E-05 |
| SERHL2 | 1.499609 | 2.827661 | 0.020925 |
| RNF151 | 1.498658 | 2.825797 | 0.018446 |
| **PPP1R15A** | 1.484046 | 2.797322 | 2.78E-05 |
| C2CD4A | 1.480473 | 2.790402 | 0.006326 |
| **NT5DC3** | 1.47029 | 2.770776 | 7.97E-05 |
| **GLRX** | 1.468183 | 2.766733 | 4.77E-06 |
| IER2 | 1.465178 | 2.760975 | 0.000502 |
| **ZNF280B** | 1.456883 | 2.745147 | 0.003017 |
| GADD45A | 1.432632 | 2.699388 | 0.000447 |
| LOC123781125 | 1.425728 | 2.686501 | 0.037831 |
| KCNE3 | 1.414026 | 2.664797 | 2.15E-07 |
| CABP4 | 1.413052 | 2.662999 | 0.02813 |
| RORA | 1.411704 | 2.660512 | 0.002822 |
| LOC123779310 | 1.409713 | 2.656844 | 1.57E-05 |
| MYO1A | 1.408897 | 2.65534 | 0.016535 |
| LOC123777310 | 1.401348 | 2.641482 | 0.036654 |
| LOC123779252 | 1.3992 | 2.637552 | 0.011025 |
| LOC123781099 | 1.399062 | 2.6373 | 0.020925 |
| LOC123778692 | 1.392637 | 2.625582 | 0.003098 |
| **ZNF594** | 1.391555 | 2.623613 | 0.006767 |
| HS3ST3B1 | 1.385512 | 2.612647 | 0.017722 |
| PRKAR1B | 1.377266 | 2.597756 | 0.031068 |
| MANEAL | 1.374344 | 2.592499 | 0.020925 |
| LOC123793256 | 1.371689 | 2.587733 | 0.033292 |
| **SPATA24** | 1.366237 | 2.577973 | 0.000525 |
| **MRPL23** | 1.363003 | 2.5722 | 4.02E-05 |
| TNFRSF9 | 1.357849 | 2.563028 | 0.000167 |
| STPG3 | 1.355952 | 2.55966 | 0.013489 |
| CATSPERG | 1.35472 | 2.557474 | 0.002533 |
| **C1QTNF4** | 1.354351 | 2.55682 | 0.008745 |
| LOC123801414 | 1.347046 | 2.543908 | 0.0037 |
| UCP3 | 1.330036 | 2.51409 | 0.010182 |
| **TEX9** | 1.31202 | 2.482889 | 0.006696 |
| **PDYN** | 1.30925 | 2.478126 | 0.028517 |
| LOC123782203 | 1.306619 | 2.473611 | 0.001339 |
| HOXC8 | 1.306066 | 2.472664 | 0.023108 |
| **BPIFC** | 1.30549 | 2.471677 | 0.033287 |
| NFIL3 | 1.302359 | 2.466318 | 0.016403 |
| **HAGHL** | 1.302079 | 2.465839 | 0.000223 |
| KIFC2 | 1.292568 | 2.449636 | 0.007099 |
| KIF26B | 1.281387 | 2.430725 | 0.001628 |
| LOC123802509 | 1.280816 | 2.429763 | 0.001433 |
| **MTHFR** | 1.277688 | 2.424502 | 8.39E-06 |
| EDA2R | 1.275252 | 2.42041 | 0.016578 |
| INHBB | 1.27293 | 2.416518 | 0.040945 |
| **MRLN** | 1.261022 | 2.396655 | 0.001259 |
| FAAP20 | 1.260162 | 2.395226 | 0.044121 |
| LOC123777272 | 1.254937 | 2.386566 | 0.041555 |
| TCIM | 1.251868 | 2.381497 | 0.001593 |
| LOC123803965 | 1.25145 | 2.380805 | 0.009308 |
| PNMA8A | 1.251392 | 2.38071 | 0.003115 |
| **GTF2IRD1** | 1.237233 | 2.357459 | 0.000393 |
| HSF4 | 1.228674 | 2.343515 | 0.00171 |
| PSD | 1.2138 | 2.319479 | 0.045805 |
| **PROCA1** | 1.211273 | 2.315418 | 0.000119 |
| LOC123776625 | 1.209258 | 2.312186 | 0.025561 |
| LOC123796494 | 1.207955 | 2.310099 | 0.013886 |
| **FGFRL1** | 1.202121 | 2.300777 | 0.002332 |
| GALNT15 | 1.195236 | 2.289824 | 0.041632 |
| LOC123780467 | 1.1876 | 2.277735 | 0.034319 |
| CUNH16orf95 | 1.181854 | 2.268682 | 0.006719 |
| LOC123784636 | 1.168952 | 2.248483 | 0.011276 |
| CCDC73 | 1.168821 | 2.248279 | 0.016652 |
| ID1 | 1.165237 | 2.242701 | 0.028517 |
| **CIR1** | 1.162255 | 2.23807 | 0.000288 |
| PAK1 | 1.160063 | 2.234672 | 0.007834 |
| **CTDSPL** | 1.15909 | 2.233165 | 0.003019 |
| EIF4EBP1 | 1.155905 | 2.22824 | 0.009308 |
| LOC123789045 | 1.153906 | 2.225155 | 0.005003 |
| CCDC106 | 1.151171 | 2.220941 | 0.017904 |
| PELI3 | 1.150464 | 2.219852 | 0.02752 |
| **PRELID2** | 1.148901 | 2.217449 | 0.00086 |
| LOC123790071 | 1.144378 | 2.210507 | 0.047686 |
| **CTSD** | 1.139049 | 2.202357 | 0.006343 |
| MRPL57 | 1.126386 | 2.183111 | 0.000647 |
| LOC123792663 | 1.125116 | 2.18119 | 0.020168 |
| **DHRS7** | 1.121302 | 2.175431 | 0.000991 |
| ARID5A | 1.11291 | 2.162815 | 0.003797 |
| SHF | 1.110966 | 2.159902 | 0.021653 |
| **SLC39A2** | 1.104109 | 2.149661 | 0.044121 |
| **BRF1** | 1.098285 | 2.141 | 0.001061 |
| PPP1R3G | 1.097168 | 2.139343 | 0.000794 |
| LOC123795619 | 1.096004 | 2.137618 | 0.021208 |
| **PTP4A3** | 1.092421 | 2.132316 | 0.015518 |
| LOC123795142 | 1.089917 | 2.128618 | 0.015356 |
| **KLHL41** | 1.085988 | 2.122829 | 0.006343 |
| RRAGD | 1.084456 | 2.120576 | 0.000734 |
| **HES1** | 1.076611 | 2.109076 | 0.01436 |
| **ZFP36** | 1.073727 | 2.104864 | 0.009159 |
| PLA2G4B | 1.057462 | 2.081267 | 0.037248 |
| **UBA52** | 1.055682 | 2.078701 | 0.004351 |
| ZSWIM9 | 1.047673 | 2.067192 | 0.044845 |
| **CBX7** | 1.045792 | 2.0645 | 0.00055 |
| **PUS7** | 1.043621 | 2.061395 | 0.001609 |
| LOC123780676 | 1.038526 | 2.054128 | 0.009942 |
| JUNB | 1.032148 | 2.045067 | 0.010593 |
| LOC123775977 | 1.031837 | 2.044626 | 0.007451 |
| **JADE2** | 1.028416 | 2.039783 | 0.004292 |
| SLC2A3 | 1.026331 | 2.036838 | 0.010576 |
| **EIF3G** | 1.02399 | 2.033535 | 0.002897 |
| MOGAT1 | 1.023193 | 2.032412 | 0.010954 |
| ARPP19 | 1.011988 | 2.016689 | 0.009789 |
| **SSR4** | 1.009902 | 2.013775 | 0.001193 |
| **SLC25A20** | 1.008014 | 2.011141 | 0.00737 |
| **JADE1** | 1.007219 | 2.010032 | 0.003574 |
| ZBTB12 | 1.004004 | 2.005559 | 0.028557 |
| LOC123775947 | 1.002954 | 2.004099 | 0.006343 |
| PLXNB1 | 1.0022 | 2.003052 | 0.016812 |
| RETREG1 | 1.001932 | 2.00268 | 0.020925 |
| USF3 | 1.000722 | 2.001001 | 0.02974 |
| MIS12 | 0.998346 | 1.997708 | 0.008382 |
| **PCSK4** | 0.994537 | 1.992441 | 0.006428 |
| **EMC10** | 0.989768 | 1.985866 | 0.002614 |
| **RPL22L1** | 0.989685 | 1.985751 | 0.001481 |
| **PELP1** | 0.983886 | 1.977786 | 0.004326 |
| SEPTIN11 | 0.981224 | 1.97414 | 0.006915 |
| CUNH11orf71 | 0.976078 | 1.96711 | 0.038896 |
| EPB41L4B | 0.974286 | 1.964669 | 0.019156 |
| **RPL30** | 0.97273 | 1.962551 | 0.009154 |
| **NEURL2** | 0.971659 | 1.961094 | 0.016399 |
| LOC123795971 | 0.971137 | 1.960385 | 0.037718 |
| PITX2 | 0.970511 | 1.959534 | 0.030192 |
| **LRRC47** | 0.966219 | 1.953713 | 0.014019 |
| LOC123804190 | 0.966016 | 1.953439 | 0.006256 |
| FRMD1 | 0.961662 | 1.947552 | 0.030495 |
| **EIF4B** | 0.960658 | 1.946197 | 0.011704 |
| RORC | 0.957174 | 1.941504 | 0.001187 |
| KYAT1 | 0.957173 | 1.941502 | 0.035306 |
| LOC123790398 | 0.957115 | 1.941424 | 0.049625 |
| **ZFP62** | 0.954276 | 1.937607 | 0.00309 |
| **CUNH12orf57** | 0.951615 | 1.934036 | 0.012893 |
| SH3RF1 | 0.951458 | 1.933826 | 0.013944 |
| ACVR2B | 0.949284 | 1.930914 | 0.010705 |
| **RPS21** | 0.946118 | 1.926681 | 0.005919 |
| **SLC2A4RG** | 0.944202 | 1.924125 | 0.004077 |
| EIF3M | 0.943248 | 1.922852 | 0.018399 |
| FOXK1 | 0.942391 | 1.92171 | 0.015382 |
| **HECA** | 0.941148 | 1.920056 | 0.010705 |
| **NDRG2** | 0.939917 | 1.918418 | 0.023902 |
| **MLLT3** | 0.937439 | 1.915125 | 0.009154 |
| **VSIG10** | 0.933463 | 1.909855 | 0.001678 |
| IER5 | 0.932285 | 1.908296 | 0.017904 |
| **RPL38** | 0.930793 | 1.906323 | 0.016658 |
| CHRAC1 | 0.925108 | 1.898827 | 0.003023 |
| KIF22 | 0.925068 | 1.898774 | 0.015259 |
| **PPP1R3E** | 0.921896 | 1.894603 | 0.013551 |
| **RPL12** | 0.921354 | 1.893892 | 0.018399 |
| **ANKRD37** | 0.921081 | 1.893534 | 0.018874 |
| KIAA1143 | 0.917939 | 1.889414 | 0.010715 |
| THSD8 | 0.917622 | 1.888999 | 0.04072 |
| LOC123795428 | 0.912732 | 1.882607 | 0.048407 |
| **ART1** | 0.910886 | 1.8802 | 0.026273 |
| PDZRN3 | 0.909908 | 1.878926 | 0.013462 |
| LOC123792876 | 0.908137 | 1.876621 | 0.034319 |
| CHRNA10 | 0.907528 | 1.875828 | 0.045851 |
| **DUSP26** | 0.905685 | 1.873434 | 0.018446 |
| BBS2 | 0.903899 | 1.871116 | 0.002053 |
| **TRMT112** | 0.902827 | 1.869726 | 0.016972 |
| **ADPRHL1** | 0.902326 | 1.869078 | 0.035306 |
| **SETD9** | 0.901594 | 1.868129 | 0.016535 |
| **P2RX6** | 0.899038 | 1.864822 | 0.005544 |
| GDAP1 | 0.898776 | 1.864484 | 0.00313 |
| WFIKKN2 | 0.891634 | 1.855276 | 0.020925 |
| LOC123785618 | 0.890404 | 1.853695 | 0.000707 |
| **GTF3A** | 0.89031 | 1.853574 | 0.0171 |
| LOC123782357 | 0.888317 | 1.851016 | 0.006312 |
| BHLHE40 | 0.886889 | 1.849184 | 0.047739 |
| **VPS9D1** | 0.883965 | 1.84544 | 0.009167 |
| **SMARCC1** | 0.883523 | 1.844875 | 0.003352 |
| LIN7B | 0.882956 | 1.84415 | 0.031412 |
| **TSPYL2** | 0.880407 | 1.840895 | 0.013969 |
| **NME4** | 0.878604 | 1.838596 | 0.01456 |
| **KIZ** | 0.878024 | 1.837856 | 0.021986 |
| DYNLT3 | 0.875377 | 1.834488 | 0.012705 |
| DUT | 0.875242 | 1.834316 | 0.031825 |
| **ZXDC** | 0.874307 | 1.833128 | 0.016756 |
| NRBF2 | 0.874181 | 1.832968 | 0.021237 |
| **ASCC1** | 0.868072 | 1.825222 | 0.016403 |
| RDH14 | 0.866617 | 1.823382 | 0.021371 |
| **ZC3H6** | 0.865234 | 1.821635 | 0.034319 |
| SMIM29 | 0.8642 | 1.82033 | 0.041082 |
| **ZFAND2B** | 0.863222 | 1.819096 | 0.022739 |
| **EIF3H** | 0.858811 | 1.813544 | 0.045107 |
| **CRAT** | 0.855893 | 1.809879 | 0.030495 |
| PABPC4 | 0.855147 | 1.808942 | 0.024441 |
| HDAC9 | 0.855029 | 1.808795 | 0.045795 |
| **RPS25** | 0.848892 | 1.801117 | 0.026826 |
| **OGFR** | 0.847619 | 1.799529 | 0.006343 |
| **IMPDH2** | 0.843861 | 1.794847 | 0.029232 |
| **ZNF70** | 0.843763 | 1.794725 | 0.042922 |
| **RPS23** | 0.841149 | 1.791476 | 0.034319 |
| **CHKB** | 0.838611 | 1.788327 | 0.021141 |
| **GLI4** | 0.836883 | 1.786187 | 0.028517 |
| **SRSF5** | 0.832955 | 1.781331 | 0.029357 |
| REPS1 | 0.832344 | 1.780576 | 0.016159 |
| **TAPT1** | 0.831777 | 1.779876 | 0.008035 |
| **FAM219B** | 0.830456 | 1.778248 | 0.003645 |
| JUN | 0.830138 | 1.777855 | 0.038404 |
| **PSMG3** | 0.828429 | 1.775751 | 0.031234 |
| **HIRIP3** | 0.827832 | 1.775016 | 0.037831 |
| **TAF9B** | 0.825827 | 1.772551 | 0.025506 |
| **P4HB** | 0.821491 | 1.767232 | 0.035635 |
| GNPTAB | 0.814337 | 1.75849 | 0.044442 |
| NUDC | 0.814024 | 1.758109 | 0.017346 |
| SHMT1 | 0.808736 | 1.751676 | 0.016624 |
| LOC123782457 | 0.807081 | 1.749667 | 0.013926 |
| CMBL | 0.793694 | 1.733508 | 0.049887 |
| LOC123778408 | 0.788411 | 1.727171 | 0.049134 |
| STK19 | 0.787427 | 1.725993 | 0.041339 |
| **TCTA** | 0.784541 | 1.722544 | 0.019819 |
| LOC123787068 | 0.784024 | 1.721927 | 0.029232 |
| **ZPR1** | 0.783759 | 1.721611 | 0.003998 |
| LOC123795266 | 0.783677 | 1.721513 | 0.034319 |
| **LGALSL** | 0.783619 | 1.721444 | 0.034319 |
| **UTP14A** | 0.780506 | 1.717733 | 0.016059 |
| **ACP2** | 0.776944 | 1.713498 | 0.015018 |
| **ANAPC16** | 0.774229 | 1.710276 | 0.005104 |
| ALKBH3 | 0.760154 | 1.693672 | 0.020934 |
| PFDN6 | 0.758119 | 1.691284 | 0.030451 |
| TPRN | 0.752395 | 1.684587 | 0.028517 |
| **SEC63** | 0.751857 | 1.683959 | 0.032819 |
| **RBM34** | 0.750067 | 1.681871 | 0.038257 |
| ARFGAP1 | 0.747056 | 1.678364 | 0.032982 |
| **KCTD2** | 0.746209 | 1.677379 | 0.026132 |
| SLX9 | 0.742088 | 1.672595 | 0.043938 |
| **SSBP2** | 0.740679 | 1.670962 | 0.016944 |
| **UBE4A** | 0.737457 | 1.667234 | 0.046734 |
| **USP4** | 0.736306 | 1.665905 | 0.04442 |
| **SUMO3** | 0.732579 | 1.661607 | 0.028388 |
| TTC33 | 0.726835 | 1.655005 | 0.017953 |
| ANAPC13 | 0.716038 | 1.642665 | 0.040558 |
| HDGFL2 | 0.714916 | 1.641387 | 0.048757 |
| **FUZ** | 0.713842 | 1.640166 | 0.048995 |
| CDK5RAP3 | 0.71122 | 1.637188 | 0.030966 |
| GBE1 | 0.710168 | 1.635994 | 0.029232 |
| LOC123790758 | 0.708919 | 1.634579 | 0.049055 |
| **RAB33B** | 0.708448 | 1.634045 | 0.036071 |
| GTPBP4 | 0.705867 | 1.631125 | 0.028517 |
| **CENPB** | 0.705478 | 1.630684 | 0.020184 |
| **RETREG2** | 0.704568 | 1.629657 | 0.013146 |
| **UBXN1** | 0.704229 | 1.629273 | 0.045107 |
| **CHMP4A** | 0.698886 | 1.62325 | 0.048757 |
| **MEPCE** | 0.698712 | 1.623055 | 0.036688 |
| PHF1 | 0.676314 | 1.598051 | 0.032819 |
| ZRANB1 | 0.628386 | 1.545834 | 0.044225 |
| MOB2 | 0.627279 | 1.544648 | 0.034319 |
| UTP18 | 0.621309 | 1.538271 | 0.041698 |
| LOC123780777 | -0.62565 | -1.54291 | 0.045357 |
| **ATXN7L3B** | -0.6703 | -1.5914 | 0.048757 |
| **SAMD11** | -0.69122 | -1.61464 | 0.040486 |
| **ZC3H7B** | -0.69423 | -1.61802 | 0.015342 |
| AOX1 | -0.70267 | -1.62752 | 0.029232 |
| **SIRT3** | -0.71457 | -1.64099 | 0.026096 |
| **GCLC** | -0.72354 | -1.65123 | 0.048407 |
| **GYS1** | -0.73 | -1.65864 | 0.048757 |
| P2RY2 | -0.73147 | -1.66033 | 0.022254 |
| **HK2** | -0.74415 | -1.67499 | 0.038203 |
| ST6GALNAC4 | -0.7504 | -1.68225 | 0.040662 |
| **BCAT2** | -0.75415 | -1.68664 | 0.028517 |
| PRXL2A | -0.765 | -1.69937 | 0.041231 |
| **PC** | -0.77738 | -1.71401 | 0.006343 |
| **MYOZ3** | -0.78193 | -1.71943 | 0.038896 |
| AQP1 | -0.78456 | -1.72257 | 0.049134 |
| ITGAV | -0.78631 | -1.72465 | 0.020184 |
| **ADAM17** | -0.78634 | -1.72469 | 0.044845 |
| **MTG1** | -0.78708 | -1.72558 | 0.020668 |
| **PLOD1** | -0.78773 | -1.72636 | 0.031825 |
| **ITIH5** | -0.7888 | -1.72763 | 0.044448 |
| MED26 | -0.79116 | -1.73046 | 0.019982 |
| NPEPL1 | -0.79321 | -1.73293 | 0.015717 |
| IDH1 | -0.79624 | -1.73657 | 0.035306 |
| SLC39A7 | -0.79981 | -1.74087 | 0.048599 |
| ANXA1 | -0.80521 | -1.74739 | 0.047389 |
| **FLRT2** | -0.81611 | -1.76066 | 0.008382 |
| THADA | -0.81905 | -1.76425 | 0.037529 |
| **TCAIM** | -0.82681 | -1.77376 | 0.010487 |
| GJA4 | -0.82705 | -1.77405 | 0.046833 |
| PDGFB | -0.82914 | -1.77662 | 0.026826 |
| TOB2 | -0.83192 | -1.78005 | 0.012705 |
| **ALDH4A1** | -0.83279 | -1.78112 | 0.016535 |
| CRIP1 | -0.83379 | -1.78236 | 0.045297 |
| TMTC1 | -0.83418 | -1.78284 | 0.020925 |
| RNF115 | -0.83448 | -1.78322 | 0.03287 |
| VSTM4 | -0.84045 | -1.79061 | 0.048757 |
| STAU2 | -0.84391 | -1.79491 | 0.016399 |
| **DBT** | -0.84543 | -1.7968 | 0.036667 |
| CHP1 | -0.84657 | -1.79821 | 0.000887 |
| MTARC1 | -0.8492 | -1.8015 | 0.031845 |
| **ATP2A3** | -0.85104 | -1.80381 | 0.047692 |
| **TRUB2** | -0.85114 | -1.80392 | 0.028154 |
| **PXMP2** | -0.8548 | -1.8085 | 0.018525 |
| MAN1A1 | -0.85483 | -1.80855 | 0.038444 |
| SNX33 | -0.85843 | -1.81307 | 0.045357 |
| **NIF3L1** | -0.85865 | -1.81334 | 0.025084 |
| **PDP1** | -0.86256 | -1.81826 | 0.043949 |
| **ECHDC1** | -0.86887 | -1.82623 | 0.025929 |
| MLEC | -0.87235 | -1.83064 | 0.02556 |
| AR | -0.87302 | -1.8315 | 0.020119 |
| G3BP2 | -0.87578 | -1.835 | 0.00643 |
| **PRDX5** | -0.87981 | -1.84013 | 0.027913 |
| LOC123785658 | -0.88661 | -1.84883 | 0.030966 |
| **PPIC** | -0.88711 | -1.84946 | 0.026826 |
| TIMP4 | -0.88727 | -1.84968 | 0.03147 |
| **PLBD1** | -0.88899 | -1.85188 | 0.005104 |
| MAP1A | -0.88996 | -1.85312 | 0.031991 |
| **EPHX2** | -0.89493 | -1.85952 | 0.040507 |
| PRKAG3 | -0.89869 | -1.86437 | 0.027607 |
| CARNS1 | -0.89887 | -1.86461 | 0.02581 |
| NAA40 | -0.89896 | -1.86472 | 0.020309 |
| **CA14** | -0.9011 | -1.86748 | 0.031845 |
| **MCCC2** | -0.90627 | -1.8742 | 0.006041 |
| **SLC29A1** | -0.90648 | -1.87446 | 0.025352 |
| ZNF169 | -0.90695 | -1.87508 | 0.007099 |
| LOC123795565 | -0.91159 | -1.88112 | 0.037831 |
| **RFTN1** | -0.91364 | -1.8838 | 0.003998 |
| **ACAD8** | -0.91452 | -1.88494 | 0.026272 |
| SPPL2B | -0.91594 | -1.8868 | 0.041487 |
| **P4HA2** | -0.91996 | -1.89207 | 0.036654 |
| ABCB8 | -0.92252 | -1.89542 | 0.020356 |
| **FITM1** | -0.92713 | -1.90149 | 0.011185 |
| **MSRA** | -0.92833 | -1.90307 | 0.008076 |
| DPP4 | -0.93371 | -1.91019 | 0.035168 |
| KCTD12 | -0.93524 | -1.91221 | 0.021981 |
| XDH | -0.93981 | -1.91827 | 0.029549 |
| MYLK | -0.93994 | -1.91845 | 0.014664 |
| **SLC37A4** | -0.9406 | -1.91932 | 0.014039 |
| ANXA2 | -0.94278 | -1.92222 | 0.017004 |
| PITX3 | -0.94391 | -1.92373 | 0.031937 |
| VLDLR | -0.94565 | -1.92605 | 0.021277 |
| **SUMF2** | -0.95097 | -1.93317 | 0.038759 |
| **HAGH** | -0.95978 | -1.94501 | 0.019154 |
| SIK2 | -0.96164 | -1.94752 | 0.020358 |
| **PDLIM1** | -0.96245 | -1.94862 | 0.030895 |
| **LAMA2** | -0.96445 | -1.95132 | 0.031068 |
| LIPA | -0.96658 | -1.95421 | 0.009154 |
| **RNF34** | -0.96666 | -1.95432 | 0.00146 |
| **TSPAN9** | -0.97712 | -1.96854 | 0.001491 |
| ADGRE5 | -0.98078 | -1.97353 | 0.036622 |
| RELT | -0.98692 | -1.98195 | 0.025495 |
| **TBC1D16** | -0.98864 | -1.98431 | 0.00734 |
| **MAP3K5** | -0.98983 | -1.98596 | 0.044121 |
| **NCAPD3** | -0.99095 | -1.9875 | 0.023091 |
| PPT1 | -0.9988 | -1.99833 | 0.001719 |
| LOC123779428 | -1.00138 | -2.00192 | 0.023699 |
| MMUT | -1.00416 | -2.00577 | 0.000445 |
| **TBC1D1** | -1.0057 | -2.00792 | 0.018378 |
| **RSAD1** | -1.00765 | -2.01064 | 0.015808 |
| **ACSS1** | -1.0113 | -2.01573 | 0.002389 |
| LOC123788946 | -1.01146 | -2.01595 | 0.020119 |
| TMEM37 | -1.01734 | -2.02418 | 0.025284 |
| **KLF13** | -1.01782 | -2.02486 | 0.005651 |
| DMTN | -1.01787 | -2.02493 | 0.03108 |
| CHRNB1 | -1.01977 | -2.02759 | 0.004629 |
| **PCSK6** | -1.02472 | -2.03456 | 0.008199 |
| **CIB1** | -1.02546 | -2.03561 | 0.030495 |
| MOB3C | -1.02893 | -2.04051 | 0.003019 |
| **ESRRB** | -1.03589 | -2.05037 | 0.02556 |
| TRIM21 | -1.03778 | -2.05306 | 0.031023 |
| **ANKRD9** | -1.03785 | -2.05316 | 0.02752 |
| **PCCB** | -1.03805 | -2.05346 | 0.005107 |
| **GPAT3** | -1.04312 | -2.06067 | 0.008745 |
| KY | -1.0512 | -2.07225 | 0.021528 |
| FGFR4 | -1.05214 | -2.07361 | 0.028904 |
| **GPT** | -1.05866 | -2.083 | 0.002068 |
| **METTL7A** | -1.06168 | -2.08737 | 0.02556 |
| PRR33 | -1.06209 | -2.08795 | 0.00732 |
| LOC123784173 | -1.06685 | -2.09486 | 0.010128 |
| **SLC38A3** | -1.06781 | -2.09625 | 0.002229 |
| VEGFD | -1.07436 | -2.10579 | 0.03147 |
| **MCCC1** | -1.07448 | -2.10596 | 0.001686 |
| LOC123786638 | -1.0784 | -2.11169 | 0.047692 |
| **ME2** | -1.09003 | -2.12878 | 0.015356 |
| CCN3 | -1.09043 | -2.12937 | 0.043045 |
| APLNR | -1.09231 | -2.13216 | 0.02392 |
| **CDK2AP2** | -1.09985 | -2.14333 | 0.001574 |
| FKBP5 | -1.10687 | -2.15378 | 0.037941 |
| PTGFRN | -1.1075 | -2.15472 | 0.020631 |
| LHFPL6 | -1.11352 | -2.16373 | 0.041197 |
| USP54 | -1.11375 | -2.16407 | 0.000688 |
| TKT | -1.11525 | -2.16632 | 0.003002 |
| **ST6GAL1** | -1.11553 | -2.16674 | 0.00374 |
| LCAT | -1.12212 | -2.17666 | 0.003574 |
| SPP2 | -1.12371 | -2.17906 | 0.049482 |
| GLIPR2 | -1.12388 | -2.17933 | 0.044845 |
| DPT | -1.13096 | -2.19005 | 0.007451 |
| RAB31 | -1.13197 | -2.19158 | 0.017824 |
| MCAM | -1.13331 | -2.19362 | 0.002614 |
| SOCS2 | -1.13412 | -2.19485 | 0.046484 |
| F2RL2 | -1.13483 | -2.19593 | 0.025413 |
| LPCAT2 | -1.13512 | -2.19638 | 0.036803 |
| CKB | -1.13534 | -2.1967 | 0.021981 |
| **TMEM176B** | -1.13634 | -2.19822 | 0.030806 |
| SLC43A2 | -1.13636 | -2.19826 | 0.014357 |
| NT5C1A | -1.14081 | -2.20504 | 0.014762 |
| CHD3 | -1.14478 | -2.21112 | 0.010663 |
| CD38 | -1.14729 | -2.21498 | 0.006343 |
| ARHGAP22 | -1.15231 | -2.2227 | 0.049275 |
| RCN3 | -1.15248 | -2.22296 | 0.033831 |
| MFAP5 | -1.15258 | -2.22311 | 0.003352 |
| **FRAS1** | -1.15314 | -2.22398 | 0.020961 |
| TGM2 | -1.15591 | -2.22825 | 0.042951 |
| SERPING1 | -1.15622 | -2.22873 | 0.010128 |
| HS3ST2 | -1.1577 | -2.23102 | 0.047279 |
| **ENO1** | -1.15787 | -2.23128 | 0.001457 |
| **PCDH1** | -1.15853 | -2.23229 | 0.001313 |
| TMC6 | -1.16133 | -2.23663 | 0.035978 |
| **ACADL** | -1.16349 | -2.23998 | 0.005475 |
| JDP2 | -1.16525 | -2.24272 | 0.033192 |
| PNMA1 | -1.17134 | -2.25221 | 0.036743 |
| VASH2 | -1.17358 | -2.25571 | 0.016263 |
| **TP53I11** | -1.17586 | -2.25927 | 0.031216 |
| SEC24D | -1.17621 | -2.25983 | 0.035532 |
| ARSA | -1.17888 | -2.264 | 0.013146 |
| FCGR2B | -1.17952 | -2.26501 | 0.039457 |
| **POLM** | -1.18122 | -2.26768 | 0.013926 |
| LOC123803841 | -1.18146 | -2.26805 | 0.043857 |
| MX1 | -1.1834 | -2.27111 | 0.004292 |
| CNTFR | -1.18486 | -2.27342 | 0.031068 |
| PLEKHF1 | -1.18535 | -2.27418 | 0.048407 |
| OLFM1 | -1.18669 | -2.27629 | 0.042858 |
| EFEMP1 | -1.18732 | -2.2773 | 0.010278 |
| CD1D | -1.1876 | -2.27774 | 0.020925 |
| ADCY7 | -1.18775 | -2.27797 | 0.021141 |
| **TST** | -1.19197 | -2.28465 | 7.45E-05 |
| HHIPL1 | -1.19832 | -2.29473 | 0.035536 |
| ABCC2 | -1.20145 | -2.2997 | 0.017979 |
| **MAP2K6** | -1.20196 | -2.30053 | 0.005644 |
| SOD3 | -1.20316 | -2.30244 | 0.008498 |
| SPSB1 | -1.20355 | -2.30306 | 0.00094 |
| ANKRD2 | -1.20399 | -2.30377 | 0.020888 |
| CEMIP2 | -1.20595 | -2.30689 | 7.78E-05 |
| **GPCPD1** | -1.20728 | -2.30902 | 0.000123 |
| ITM2A | -1.21078 | -2.31463 | 0.025697 |
| **MPST** | -1.21742 | -2.3253 | 7.67E-05 |
| SLC22A17 | -1.21903 | -2.32789 | 0.018378 |
| **MTFP1** | -1.21929 | -2.32833 | 0.016905 |
| TMEM233 | -1.22056 | -2.33038 | 0.002479 |
| LTBP1 | -1.2217 | -2.33221 | 0.007331 |
| SELENBP1 | -1.22279 | -2.33398 | 0.002354 |
| CHRD | -1.23054 | -2.34655 | 0.031573 |
| **STK32C** | -1.23341 | -2.35121 | 0.017087 |
| LGI2 | -1.23518 | -2.35411 | 0.049213 |
| **COL4A2** | -1.23755 | -2.35797 | 0.011472 |
| **IGFBP7** | -1.24044 | -2.36271 | 0.003613 |
| **BCKDHB** | -1.24496 | -2.37012 | 0.000736 |
| **STAB1** | -1.24567 | -2.37128 | 0.011722 |
| **LAMB1** | -1.24679 | -2.37313 | 0.014664 |
| PHGDH | -1.2483 | -2.37561 | 0.026862 |
| LHFPL2 | -1.25027 | -2.37886 | 0.008754 |
| VCAM1 | -1.2511 | -2.38024 | 0.015243 |
| MRGPRF | -1.2531 | -2.38353 | 0.042858 |
| **SAMHD1** | -1.26051 | -2.39581 | 0.000324 |
| SULF1 | -1.26054 | -2.39585 | 0.017644 |
| LOC123789134 | -1.26134 | -2.39718 | 0.047938 |
| FERMT3 | -1.26202 | -2.39832 | 0.016658 |
| OLIG1 | -1.26244 | -2.39901 | 0.005966 |
| CCDC170 | -1.26546 | -2.40404 | 0.046882 |
| TP53INP2 | -1.26551 | -2.40412 | 0.002233 |
| **HSPB1** | -1.26876 | -2.40955 | 0.007338 |
| SCX | -1.27138 | -2.41393 | 0.038404 |
| LPAR1 | -1.27364 | -2.41771 | 0.043257 |
| DCN | -1.27605 | -2.42174 | 0.008035 |
| GSN | -1.27665 | -2.42275 | 0.001203 |
| TMED3 | -1.28103 | -2.43013 | 0.038759 |
| P2RY14 | -1.28128 | -2.43054 | 0.02677 |
| **MLXIPL** | -1.28237 | -2.43238 | 0.00198 |
| **OSGIN1** | -1.28336 | -2.43405 | 0.030495 |
| **MLF1** | -1.28377 | -2.43475 | 0.028557 |
| CCND2 | -1.2848 | -2.43648 | 0.002835 |
| CKAP4 | -1.28491 | -2.43666 | 0.01595 |
| SMOC2 | -1.28663 | -2.43957 | 0.035928 |
| LOC123779547 | -1.28719 | -2.44053 | 0.04442 |
| COTL1 | -1.28767 | -2.44134 | 0.001943 |
| ETV1 | -1.29057 | -2.44625 | 0.048757 |
| SMOC1 | -1.29458 | -2.45305 | 0.010993 |
| COL18A1 | -1.29459 | -2.45307 | 0.00558 |
| KAZALD1 | -1.2971 | -2.45734 | 0.048757 |
| LOC123792682 | -1.29815 | -2.45913 | 0.020925 |
| RSPO3 | -1.29837 | -2.45952 | 0.01595 |
| **MAP1LC3A** | -1.29931 | -2.46111 | 8.96E-05 |
| LOC123799110 | -1.29969 | -2.46175 | 2.25E-06 |
| CYS1 | -1.30214 | -2.46595 | 0.018316 |
| LYVE1 | -1.30593 | -2.47243 | 0.006229 |
| **GALNS** | -1.31056 | -2.48037 | 0.002296 |
| LGALS9 | -1.31113 | -2.48137 | 0.010705 |
| **NEK3** | -1.31477 | -2.48763 | 0.045795 |
| PDLIM4 | -1.31489 | -2.48783 | 0.041082 |
| **ACOT11** | -1.31978 | -2.49628 | 0.001609 |
| **HEG1** | -1.32048 | -2.4975 | 0.00397 |
| GPR153 | -1.32078 | -2.498 | 0.015243 |
| CSF1R | -1.32601 | -2.50709 | 0.005588 |
| CASQ2 | -1.32946 | -2.51309 | 0.000629 |
| **HSPG2** | -1.33044 | -2.51479 | 0.010705 |
| DMGDH | -1.33233 | -2.51808 | 0.033831 |
| LOC123781498 | -1.33633 | -2.52508 | 0.046484 |
| LOC123792080 | -1.33893 | -2.52963 | 0.006647 |
| LOC123792712 | -1.34337 | -2.53743 | 0.02288 |
| **NID1** | -1.34495 | -2.54021 | 0.002227 |
| **ARHGEF28** | -1.34537 | -2.54096 | 0.002312 |
| OLFM2 | -1.34609 | -2.54223 | 0.035306 |
| **CLIP4** | -1.3478 | -2.54523 | 0.000127 |
| CNTN1 | -1.34853 | -2.54652 | 0.029527 |
| CNTN4 | -1.35196 | -2.55259 | 0.026096 |
| ADGRG6 | -1.35392 | -2.55606 | 0.021698 |
| FBLN1 | -1.35472 | -2.55748 | 0.000689 |
| SYT12 | -1.35649 | -2.56061 | 0.045258 |
| TMEM229B | -1.36009 | -2.56701 | 0.02386 |
| RASSF5 | -1.36188 | -2.57019 | 0.031346 |
| F13A1 | -1.36445 | -2.57478 | 0.008869 |
| LOC123785714 | -1.36583 | -2.57724 | 0.008562 |
| RECK | -1.36718 | -2.57966 | 0.015525 |
| FAM151A | -1.37246 | -2.58912 | 0.007099 |
| CAPN6 | -1.37277 | -2.58967 | 0.003436 |
| **PHOSPHO1** | -1.37539 | -2.59439 | 1.05E-06 |
| LOC123786770 | -1.37919 | -2.60122 | 0.046882 |
| LOC123776422 | -1.38148 | -2.60536 | 0.000186 |
| LRP1 | -1.38172 | -2.60579 | 0.022221 |
| **SLC25A33** | -1.38631 | -2.61409 | 0.002443 |
| **ERGIC1** | -1.38661 | -2.61464 | 0.00247 |
| ALG14 | -1.39059 | -2.62185 | 0.009528 |
| PAPSS2 | -1.39083 | -2.62229 | 0.039107 |
| PHETA2 | -1.39098 | -2.62257 | 0.013961 |
| LOC123795557 | -1.39209 | -2.62459 | 0.038404 |
| SERPINH1 | -1.39441 | -2.62882 | 0.005722 |
| LOC123800768 | -1.39597 | -2.63166 | 0.002045 |
| TRNP1 | -1.39598 | -2.63168 | 0.005865 |
| **PYROXD2** | -1.39617 | -2.63202 | 0.002435 |
| **LRRC36** | -1.39847 | -2.63623 | 0.041487 |
| DIS3L2 | -1.40158 | -2.64191 | 2.83E-05 |
| LOC123797070 | -1.40285 | -2.64423 | 0.001893 |
| **DAGLA** | -1.40322 | -2.64491 | 0.000186 |
| **GPM6B** | -1.40567 | -2.64941 | 0.009038 |
| **HIGD1B** | -1.40592 | -2.64987 | 0.02342 |
| LOC123793442 | -1.40644 | -2.65082 | 3.2E-05 |
| LOC123779629 | -1.40822 | -2.65409 | 0.033765 |
| NTN4 | -1.40882 | -2.6552 | 0.000947 |
| IGFBP5 | -1.41867 | -2.67338 | 0.024393 |
| CLU | -1.42052 | -2.67681 | 0.016399 |
| **COLGALT2** | -1.42377 | -2.68286 | 0.042858 |
| CEP72 | -1.42386 | -2.68302 | 0.017841 |
| **PIGZ** | -1.42524 | -2.68559 | 0.014664 |
| CIDEA | -1.4258 | -2.68663 | 0.020614 |
| FCER1G | -1.42837 | -2.69142 | 0.010715 |
| G0S2 | -1.43138 | -2.69704 | 0.042858 |
| COL4A5 | -1.43461 | -2.70308 | 0.023108 |
| DBN1 | -1.43526 | -2.70431 | 0.004462 |
| P3H3 | -1.43541 | -2.70459 | 0.009308 |
| LOC123800772 | -1.4376 | -2.7087 | 0.020309 |
| ISLR | -1.43769 | -2.70886 | 0.000121 |
| LOC123795184 | -1.43847 | -2.71033 | 0.030895 |
| **AGBL1** | -1.44599 | -2.7245 | 0.045225 |
| C1QA | -1.44975 | -2.73161 | 0.037598 |
| TAGLN3 | -1.45019 | -2.73245 | 0.029001 |
| BAIAP2L1 | -1.45085 | -2.73369 | 0.002084 |
| GDPD5 | -1.45178 | -2.73545 | 0.049813 |
| MMP14 | -1.453 | -2.73778 | 0.037284 |
| PMEPA1 | -1.45814 | -2.74755 | 6.74E-05 |
| SCPEP1 | -1.45923 | -2.74962 | 7.47E-06 |
| GK | -1.46125 | -2.75347 | 0.000258 |
| KLHL25 | -1.46155 | -2.75405 | 0.002579 |
| LOC123778488 | -1.46323 | -2.75725 | 0.021981 |
| LAMA4 | -1.46622 | -2.76297 | 0.001196 |
| ADAP1 | -1.46924 | -2.76875 | 0.016471 |
| MYOG | -1.47454 | -2.77895 | 4.59E-05 |
| **RGS3** | -1.47593 | -2.78163 | 7.63E-07 |
| **PCSK5** | -1.47595 | -2.78167 | 0.02812 |
| FMN2 | -1.47654 | -2.7828 | 0.028517 |
| ECM2 | -1.48067 | -2.79078 | 0.001481 |
| SATB2 | -1.48349 | -2.79624 | 0.029663 |
| PTGS1 | -1.48386 | -2.79696 | 0.000445 |
| ECM1 | -1.48398 | -2.7972 | 0.000217 |
| ARSJ | -1.48432 | -2.79786 | 0.047114 |
| OTUD7A | -1.48934 | -2.8076 | 0.005223 |
| MEDAG | -1.49238 | -2.81353 | 0.001543 |
| NCF4 | -1.49562 | -2.81986 | 0.041861 |
| VWA5A | -1.49776 | -2.82404 | 0.000267 |
| MZB1 | -1.49827 | -2.82503 | 0.020309 |
| ITGA8 | -1.49946 | -2.82736 | 0.005475 |
| ARHGAP44 | -1.50092 | -2.83022 | 0.014664 |
| EPHA3 | -1.50228 | -2.8329 | 0.040335 |
| LOC123793587 | -1.50423 | -2.83674 | 0.015958 |
| LOC123802467 | -1.50731 | -2.8428 | 0.047537 |
| GAS7 | -1.50739 | -2.84295 | 0.004297 |
| ISLR2 | -1.50888 | -2.84589 | 0.003471 |
| LOC123793247 | -1.51609 | -2.86015 | 0.034474 |
| **MFSD4B** | -1.51737 | -2.86269 | 0.003187 |
| LOC123803845 | -1.51938 | -2.86668 | 0.001857 |
| LOC123788434 | -1.52036 | -2.86862 | 0.003745 |
| LOC123802199 | -1.52169 | -2.87127 | 0.034319 |
| CACHD1 | -1.52455 | -2.87698 | 0.006251 |
| LOC123776924 | -1.52761 | -2.88308 | 0.012392 |
| LOC123787489 | -1.52862 | -2.88509 | 0.019052 |
| LOC123780817 | -1.52987 | -2.88759 | 0.029549 |
| SLIT3 | -1.53088 | -2.88962 | 0.009081 |
| ASNS | -1.53103 | -2.88993 | 0.018505 |
| TGFBI | -1.53309 | -2.89406 | 0.00102 |
| NFASC | -1.53379 | -2.89545 | 0.022242 |
| LOC123785906 | -1.534 | -2.89587 | 0.000164 |
| LOC123785559 | -1.53434 | -2.89655 | 0.045795 |
| MASP1 | -1.53843 | -2.90477 | 0.006849 |
| NINJ2 | -1.54646 | -2.92099 | 0.014501 |
| CDC42EP3 | -1.54695 | -2.92199 | 1.19E-09 |
| C1QTNF2 | -1.54711 | -2.92231 | 0.006343 |
| MYL9 | -1.55392 | -2.93613 | 3.84E-05 |
| SDK1 | -1.55763 | -2.9437 | 0.026447 |
| **ARSI** | -1.55779 | -2.94403 | 0.008163 |
| IGF1 | -1.55789 | -2.94423 | 0.000153 |
| IRX3 | -1.55841 | -2.94529 | 0.013926 |
| AHNAK2 | -1.55924 | -2.94699 | 0.02386 |
| DNM1 | -1.56133 | -2.95126 | 0.017352 |
| LOC123800105 | -1.56149 | -2.95159 | 0.015557 |
| TLDC2 | -1.56434 | -2.95742 | 0.000484 |
| **ST8SIA5** | -1.56806 | -2.96506 | 0.000497 |
| CAMK1D | -1.57471 | -2.97876 | 0.02813 |
| **COL4A4** | -1.5762 | -2.98183 | 0.002279 |
| **GLUL** | -1.57673 | -2.98294 | 0.000108 |
| PTGES | -1.57713 | -2.98375 | 0.036622 |
| LOC123786017 | -1.58234 | -2.99455 | 0.002691 |
| HOXA13 | -1.58253 | -2.99496 | 0.008418 |
| SQLE | -1.58293 | -2.99578 | 0.038759 |
| BMP7 | -1.58384 | -2.99767 | 0.012581 |
| PLD5 | -1.5843 | -2.99863 | 0.012498 |
| SCARA5 | -1.58515 | -3.00038 | 0.000319 |
| LGI4 | -1.58574 | -3.00161 | 0.020184 |
| OAF | -1.58675 | -3.00372 | 0.001125 |
| SLCO2A1 | -1.58798 | -3.00629 | 0.008432 |
| SCIN | -1.59256 | -3.01584 | 0.034319 |
| NALCN | -1.59379 | -3.0184 | 0.011322 |
| LOXL3 | -1.59394 | -3.01873 | 0.016652 |
| CRISPLD2 | -1.59827 | -3.0278 | 2.51E-05 |
| EMILIN1 | -1.60218 | -3.03603 | 0.000162 |
| **PODN** | -1.61545 | -3.06407 | 6.71E-05 |
| CTSH | -1.61607 | -3.06538 | 0.000583 |
| SVEP1 | -1.61615 | -3.06556 | 0.002712 |
| LDLR | -1.62121 | -3.07633 | 0.001574 |
| PLXDC1 | -1.62598 | -3.08653 | 0.00015 |
| LGI3 | -1.63364 | -3.10295 | 0.003741 |
| IL1R2 | -1.63504 | -3.10596 | 0.020072 |
| SLC45A2 | -1.63515 | -3.10619 | 0.020168 |
| GLIPR1 | -1.63553 | -3.10702 | 0.000565 |
| CD276 | -1.63688 | -3.10992 | 0.003838 |
| PXDNL | -1.63769 | -3.11168 | 0.012637 |
| CRYM | -1.63814 | -3.11263 | 0.007059 |
| ADAMTS12 | -1.64228 | -3.12159 | 0.031845 |
| PDGFRL | -1.64947 | -3.13719 | 0.010663 |
| PLA2G7 | -1.65647 | -3.15245 | 0.047537 |
| TNS3 | -1.65724 | -3.15412 | 0.012328 |
| EYA2 | -1.66216 | -3.1649 | 0.024867 |
| FREM2 | -1.66259 | -3.16585 | 5.96E-05 |
| CADM3 | -1.66269 | -3.16605 | 0.003023 |
| GAL3ST4 | -1.6645 | -3.17005 | 0.028267 |
| IRF5 | -1.6662 | -3.17379 | 0.04942 |
| MSC | -1.67131 | -3.18503 | 0.00767 |
| ARMC9 | -1.67202 | -3.18662 | 0.000445 |
| FBLN2 | -1.67218 | -3.18697 | 4.8E-05 |
| TNFRSF8 | -1.67316 | -3.18913 | 0.019847 |
| CCDC160 | -1.67533 | -3.19392 | 0.011522 |
| ACP5 | -1.67577 | -3.19491 | 0.03406 |
| MATN2 | -1.67602 | -3.19545 | 0.002897 |
| ELN | -1.677 | -3.19763 | 0.000784 |
| **EGF** | -1.68028 | -3.2049 | 5.61E-05 |
| **PHKG1** | -1.68299 | -3.21093 | 8.96E-05 |
| C1QB | -1.68403 | -3.21325 | 0.038469 |
| **ELOVL5** | -1.69128 | -3.22944 | 4.38E-05 |
| CD44 | -1.69273 | -3.23268 | 0.00018 |
| SMPDL3B | -1.69446 | -3.23656 | 1.44E-05 |
| THBS2 | -1.69725 | -3.24282 | 0.031845 |
| FSTL1 | -1.70211 | -3.25375 | 0.000108 |
| WFDC1 | -1.70386 | -3.25772 | 0.001816 |
| **EXTL1** | -1.70537 | -3.26112 | 2.34E-05 |
| LOC123788950 | -1.70539 | -3.26117 | 0.006215 |
| LOC123784598 | -1.70851 | -3.26823 | 0.00092 |
| COL6A6 | -1.71061 | -3.27298 | 0.00463 |
| IGFBP4 | -1.71466 | -3.2822 | 0.000185 |
| **NID2** | -1.71769 | -3.28909 | 0.000542 |
| FADS2 | -1.72097 | -3.29658 | 0.000295 |
| NIPSNAP1 | -1.72321 | -3.30169 | 0.01377 |
| LOC123788867 | -1.73851 | -3.3369 | 0.006307 |
| ENPP2 | -1.74006 | -3.34049 | 0.000488 |
| RRBP1 | -1.74522 | -3.35246 | 0.001131 |
| IGFBP2 | -1.74692 | -3.3564 | 0.015022 |
| DCLK1 | -1.74701 | -3.35663 | 0.003825 |
| CDH11 | -1.75525 | -3.37584 | 0.009154 |
| THY1 | -1.76023 | -3.38753 | 4.11E-05 |
| LOC123777490 | -1.76049 | -3.38812 | 0.004292 |
| C1R | -1.76252 | -3.39291 | 0.001733 |
| CDO1 | -1.76812 | -3.4061 | 0.015022 |
| SLC22A4 | -1.76884 | -3.40779 | 1.29E-05 |
| HSD11B2 | -1.77264 | -3.41679 | 0.015461 |
| **CFAP410** | -1.77409 | -3.42023 | 0.004351 |
| MXRA5 | -1.78035 | -3.4351 | 0.007346 |
| ACKR2 | -1.78046 | -3.43535 | 0.0016 |
| GPNMB | -1.78048 | -3.43541 | 0.001524 |
| LOC123781335 | -1.78081 | -3.43619 | 0.000115 |
| **ABAT** | -1.78201 | -3.43906 | 5.86E-06 |
| GLDN | -1.78256 | -3.44037 | 0.003023 |
| LOC123796735 | -1.78548 | -3.44733 | 0.00071 |
| ADCYAP1R1 | -1.78714 | -3.4513 | 0.006775 |
| APOD | -1.78778 | -3.45283 | 0.016059 |
| PLEKHA7 | -1.7878 | -3.45288 | 0.025393 |
| FLRT3 | -1.78891 | -3.45553 | 0.023829 |
| FAM180B | -1.79061 | -3.45962 | 0.00506 |
| CD109 | -1.79209 | -3.46317 | 0.008035 |
| HAPLN3 | -1.79341 | -3.46633 | 0.007309 |
| FOLR2 | -1.79375 | -3.46716 | 7.91E-05 |
| CX3CR1 | -1.79873 | -3.47913 | 0.007863 |
| AOAH | -1.80114 | -3.48496 | 0.012133 |
| BMP1 | -1.80306 | -3.4896 | 0.000618 |
| C1QC | -1.80404 | -3.49196 | 0.028904 |
| **SYN1** | -1.8057 | -3.49598 | 1.44E-07 |
| **PLK5** | -1.80735 | -3.5 | 3.97E-06 |
| COL12A1 | -1.80839 | -3.50251 | 0.002598 |
| FAM20A | -1.80888 | -3.50371 | 0.013999 |
| LOC123788969 | -1.81612 | -3.52133 | 0.005193 |
| LOC123798248 | -1.8199 | -3.53058 | 0.012664 |
| ITM2C | -1.82099 | -3.53323 | 0.005109 |
| SCN7A | -1.83133 | -3.55864 | 0.030495 |
| LOC123779418 | -1.83276 | -3.56219 | 0.005564 |
| ACTG2 | -1.83504 | -3.56782 | 0.007552 |
| LOC123778097 | -1.84483 | -3.59211 | 0.00901 |
| CHPF | -1.84662 | -3.59658 | 0.004668 |
| FAM174B | -1.84868 | -3.6017 | 0.005502 |
| IGFBP6 | -1.85225 | -3.61063 | 6.54E-06 |
| TP53I3 | -1.85754 | -3.6239 | 0.000107 |
| CTHRC1 | -1.85805 | -3.62516 | 0.023339 |
| CPXM2 | -1.85818 | -3.62551 | 0.003019 |
| NGFR | -1.8629 | -3.63738 | 0.015525 |
| LOC123803887 | -1.86395 | -3.64003 | 0.022553 |
| CRHR2 | -1.86883 | -3.65236 | 4.09E-06 |
| COL6A1 | -1.88242 | -3.68692 | 9.53E-05 |
| LOC123783598 | -1.88518 | -3.69399 | 0.000171 |
| ROBO1 | -1.88611 | -3.69636 | 0.000236 |
| ALDH1L2 | -1.8909 | -3.70867 | 0.010761 |
| ICAM3 | -1.89705 | -3.72451 | 0.020309 |
| GPC6 | -1.89886 | -3.72919 | 0.002137 |
| GRIA3 | -1.89972 | -3.73142 | 0.01589 |
| AEBP1 | -1.90773 | -3.75219 | 0.004503 |
| **COL15A1** | -1.90811 | -3.75316 | 5.93E-05 |
| RUNX2 | -1.9085 | -3.75418 | 0.002233 |
| SGK2 | -1.91048 | -3.75934 | 0.016744 |
| KERA | -1.91137 | -3.76167 | 0.000766 |
| ENTPD2 | -1.92115 | -3.78726 | 3.44E-05 |
| **COL4A1** | -1.9218 | -3.78895 | 2.53E-05 |
| QPCT | -1.93132 | -3.81403 | 0.004181 |
| **ADAMTSL2** | -1.93662 | -3.82808 | 0.000285 |
| LOC123785735 | -1.94049 | -3.83836 | 0.002233 |
| VCAN | -1.94137 | -3.8407 | 5.24E-07 |
| **DPYSL3** | -1.94368 | -3.84685 | 8.64E-11 |
| ADORA1 | -1.94381 | -3.8472 | 0.001526 |
| LOC123794537 | -1.94428 | -3.84847 | 0.006009 |
| CIDEC | -1.94726 | -3.85642 | 0.003333 |
| LOC123784105 | -1.94882 | -3.8606 | 5.76E-05 |
| BGN | -1.95243 | -3.87025 | 4.02E-05 |
| NMB | -1.95493 | -3.87697 | 7.48E-07 |
| **HMCN2** | -1.9602 | -3.89116 | 4E-06 |
| **BEST3** | -1.96085 | -3.89291 | 0.000305 |
| COL6A3 | -1.97049 | -3.91901 | 0.00055 |
| ACACA | -1.97588 | -3.93369 | 1.12E-05 |
| CD209 | -1.97673 | -3.93601 | 0.000408 |
| VAT1L | -1.97937 | -3.94322 | 0.001678 |
| THSD4 | -1.97956 | -3.94373 | 0.001728 |
| LRRC25 | -1.98148 | -3.94897 | 0.001086 |
| JCHAIN | -1.98152 | -3.9491 | 0.011149 |
| FKBP10 | -1.98156 | -3.9492 | 0.001893 |
| LOC123803241 | -1.9823 | -3.95123 | 0.002233 |
| FANK1 | -1.98292 | -3.95293 | 0.010805 |
| ILDR2 | -1.98454 | -3.95737 | 0.010705 |
| COL6A2 | -1.98637 | -3.96238 | 0.000159 |
| MCHR1 | -1.99153 | -3.97657 | 0.000295 |
| **CTH** | -1.99818 | -3.99496 | 0.002105 |
| ANGPTL2 | -2.00287 | -4.00798 | 1.38E-05 |
| ADAMTS7 | -2.00785 | -4.02181 | 0.00294 |
| GJC3 | -2.00856 | -4.02381 | 0.041861 |
| GLI1 | -2.01044 | -4.02905 | 0.024969 |
| EPHB2 | -2.01321 | -4.0368 | 0.021842 |
| CHI3L1 | -2.01964 | -4.05481 | 0.034319 |
| LOC123781399 | -2.03499 | -4.0982 | 0.001125 |
| EPN3 | -2.04834 | -4.1363 | 0.000253 |
| THBS3 | -2.05306 | -4.14985 | 2.49E-05 |
| C4A | -2.05847 | -4.16543 | 0.016905 |
| EMILIN2 | -2.05991 | -4.1696 | 0.000223 |
| MMP2 | -2.07608 | -4.21661 | 0.000576 |
| OGN | -2.08656 | -4.24733 | 0.000794 |
| FGF6 | -2.0869 | -4.24835 | 0.000598 |
| ADAMTS17 | -2.08892 | -4.25429 | 0.006099 |
| TPBG | -2.08894 | -4.25434 | 0.003825 |
| **ACSL6** | -2.09561 | -4.27405 | 0.004227 |
| LOC123781330 | -2.1025 | -4.29452 | 2.65E-05 |
| **COL4A3** | -2.10349 | -4.29747 | 0.000536 |
| HIPK4 | -2.10848 | -4.31236 | 0.004668 |
| LOC123796571 | -2.11656 | -4.33659 | 0.002045 |
| LUM | -2.11842 | -4.34217 | 6.03E-08 |
| EMID1 | -2.12435 | -4.36006 | 0.020309 |
| LOC123783924 | -2.12632 | -4.36601 | 0.001061 |
| **GADL1** | -2.12984 | -4.37669 | 3.68E-07 |
| **ACSM5** | -2.14281 | -4.41622 | 9.83E-06 |
| LOC123800527 | -2.14366 | -4.41882 | 0.000582 |
| MMP16 | -2.15628 | -4.45764 | 0.006202 |
| LOC123781779 | -2.16026 | -4.46996 | 1.96E-05 |
| SARDH | -2.16304 | -4.47857 | 0.000848 |
| LOC123790157 | -2.16725 | -4.49166 | 0.006343 |
| SORCS1 | -2.1706 | -4.50212 | 0.005722 |
| LGR5 | -2.17478 | -4.51517 | 0.025316 |
| MFSD4A | -2.18141 | -4.53595 | 0.004338 |
| **PI15** | -2.19164 | -4.56826 | 0.000629 |
| ASPN | -2.20319 | -4.60496 | 0.000107 |
| CHAD | -2.2054 | -4.61202 | 0.017141 |
| PLP1 | -2.21528 | -4.64371 | 0.022677 |
| CD248 | -2.22191 | -4.66511 | 1.49E-06 |
| SFRP5 | -2.23666 | -4.71303 | 0.017352 |
| RET | -2.23842 | -4.7188 | 0.000124 |
| SPRN | -2.24359 | -4.73574 | 0.001135 |
| FER1L6 | -2.24643 | -4.74506 | 5.96E-05 |
| MFAP2 | -2.24758 | -4.74886 | 0.000302 |
| LOX | -2.25582 | -4.77605 | 0.000422 |
| KCTD14 | -2.26894 | -4.81969 | 0.000529 |
| LOC123802316 | -2.2706 | -4.82523 | 0.01266 |
| LOC123780105 | -2.27126 | -4.82745 | 0.015525 |
| CSMD2 | -2.27286 | -4.8328 | 0.006416 |
| **DHTKD1** | -2.27806 | -4.85024 | 2.04E-08 |
| TNNT2 | -2.27846 | -4.85161 | 0.001816 |
| TNFAIP6 | -2.28148 | -4.86175 | 0.002209 |
| **DHCR24** | -2.30155 | -4.92987 | 0.000403 |
| EBF4 | -2.30853 | -4.95379 | 0.002443 |
| SRPX | -2.30898 | -4.95531 | 0.001897 |
| LOC123776927 | -2.31057 | -4.96078 | 0.000235 |
| TNFAIP8L3 | -2.32258 | -5.00224 | 0.012581 |
| SBSPON | -2.32667 | -5.01645 | 0.035306 |
| TMEM119 | -2.33575 | -5.04813 | 0.001085 |
| **PTGR1** | -2.34959 | -5.09679 | 6.36E-06 |
| FBN1 | -2.35154 | -5.10367 | 1.44E-07 |
| TENT5B | -2.35587 | -5.11902 | 2.8E-05 |
| UNC80 | -2.36443 | -5.1495 | 0.002635 |
| SSC5D | -2.36497 | -5.15141 | 6.44E-05 |
| C1S | -2.36516 | -5.15208 | 0.000223 |
| SDC1 | -2.36564 | -5.1538 | 0.003471 |
| PPP2R2C | -2.37054 | -5.17134 | 4.61E-05 |
| FOXO6 | -2.39922 | -5.27517 | 2.4E-07 |
| MMP19 | -2.40449 | -5.29449 | 8.2E-06 |
| SPON2 | -2.41559 | -5.33538 | 3.65E-05 |
| COL5A2 | -2.41841 | -5.3458 | 7.55E-06 |
| FAM180A | -2.42271 | -5.36176 | 0.008828 |
| ITGA11 | -2.43655 | -5.41347 | 6.46E-05 |
| SLC1A2 | -2.43928 | -5.42371 | 0.000322 |
| TTC7A | -2.4423 | -5.43509 | 6.23E-09 |
| **SLC22A3** | -2.4488 | -5.45962 | 0 |
| **KIF23** | -2.45965 | -5.50084 | 0.000302 |
| GREM2 | -2.47016 | -5.54107 | 4.71E-05 |
| SLC6A4 | -2.47605 | -5.56371 | 0.002614 |
| SEMA3D | -2.47768 | -5.56999 | 0.009308 |
| LOC123783617 | -2.47898 | -5.57502 | 0.00737 |
| LOC123785045 | -2.48197 | -5.58658 | 0.034419 |
| C1QTNF1 | -2.49106 | -5.62193 | 1.47E-05 |
| THBS4 | -2.49741 | -5.64671 | 2.86E-09 |
| TEDC1 | -2.50316 | -5.66926 | 0.000596 |
| BPI | -2.50919 | -5.69301 | 0.007863 |
| **ALDH1L1** | -2.52007 | -5.73609 | 1.09E-09 |
| MRC2 | -2.52712 | -5.76419 | 6.88E-10 |
| KCNA1 | -2.54403 | -5.83216 | 1.46E-07 |
| SLC2A1 | -2.55638 | -5.88228 | 0.003542 |
| PCOLCE | -2.57421 | -5.95545 | 7.37E-07 |
| PIEZO2 | -2.57495 | -5.9585 | 0.006306 |
| FN1 | -2.58078 | -5.98265 | 1.06E-05 |
| TIMD4 | -2.59908 | -6.05901 | 0.000916 |
| SPARC | -2.60351 | -6.07764 | 4.38E-05 |
| **FADS1** | -2.61231 | -6.11484 | 1.38E-05 |
| IGSF23 | -2.62709 | -6.17779 | 1.62E-07 |
| LOC123799727 | -2.64583 | -6.25857 | 0.008046 |
| ADAM12 | -2.65089 | -6.28053 | 0.000629 |
| FNDC1 | -2.65442 | -6.29595 | 0.000107 |
| SLC14A2 | -2.67286 | -6.37691 | 3.49E-05 |
| MGST1 | -2.69837 | -6.49068 | 9.92E-05 |
| **AASS** | -2.75137 | -6.73355 | 7.44E-10 |
| ADAMTS16 | -2.75636 | -6.75691 | 0.000285 |
| C1QTNF3 | -2.7814 | -6.8752 | 5.86E-06 |
| **DGAT2** | -2.80241 | -6.97605 | 0 |
| ISM1 | -2.80445 | -6.98594 | 0.000127 |
| FAM43B | -2.81087 | -7.01709 | 2.93E-05 |
| COL8A1 | -2.81362 | -7.03046 | 2.49E-08 |
| LOC123799055 | -2.84341 | -7.17716 | 4.38E-05 |
| GFRA3 | -2.86518 | -7.28625 | 0.000481 |
| SLC4A8 | -2.86811 | -7.30109 | 2.16E-05 |
| **INHA** | -2.87962 | -7.35954 | 4.38E-05 |
| COL14A1 | -2.88023 | -7.36265 | 6.93E-07 |
| FBLN7 | -2.88444 | -7.38422 | 0.000122 |
| LOC123782854 | -2.88663 | -7.39543 | 1.37E-07 |
| **FASN** | -2.8891 | -7.4081 | 2.89E-12 |
| COL5A1 | -2.91141 | -7.52355 | 8.11E-06 |
| LOXL2 | -2.9207 | -7.57213 | 1E-07 |
| FMOD | -2.96182 | -7.79107 | 2.55E-05 |
| GDF10 | -2.96495 | -7.80798 | 7.03E-05 |
| LOC123793510 | -2.9754 | -7.86476 | 0.025561 |
| ELOVL6 | -3.00626 | -8.03479 | 1.91E-05 |
| LOC123790105 | -3.06392 | -8.36244 | 1.24E-07 |
| LOC123802793 | -3.10278 | -8.59073 | 1.49E-06 |
| ITGBL1 | -3.11823 | -8.68322 | 1.05E-05 |
| LOC123788954 | -3.12793 | -8.7418 | 8.44E-07 |
| C2 | -3.14602 | -8.85209 | 1.51E-06 |
| MMP23B | -3.1633 | -8.95877 | 3.55E-05 |
| PSAT1 | -3.1684 | -8.99052 | 0.000299 |
| TNC | -3.22262 | -9.33478 | 6.03E-08 |
| BTBD11 | -3.23028 | -9.38452 | 3.14E-10 |
| CCDC80 | -3.23171 | -9.39382 | 1.22E-08 |
| LOC123804251 | -3.31976 | -9.98496 | 1.19E-09 |
| **SERPINF1** | -3.32018 | -9.98789 | 1.83E-09 |
| SFRP4 | -3.39513 | -10.5205 | 3.36E-06 |
| SPON1 | -3.41223 | -10.646 | 0.000996 |
| CPXM1 | -3.48386 | -11.1879 | 4.9E-05 |
| LOC123793509 | -3.48722 | -11.214 | 1.07E-08 |
| **OXCT1** | -3.67558 | -12.7779 | 0 |
| LOC123789276 | -3.89459 | -14.8727 | 4.49E-07 |
| POSTN | -3.96296 | -15.5945 | 6.03E-08 |
| MYBPH | -4.14514 | -17.6934 | 0 |
| LOC123802229 | -4.27861 | -19.4085 | 0 |
| CREB3L1 | -4.37446 | -20.7417 | 6.03E-08 |
| NUP62CL | -4.40404 | -21.1713 | 3.01E-11 |
| ADAMTS2 | -4.51173 | -22.8122 | 6.53E-12 |
| MTHFD2 | -4.58605 | -24.0181 | 5.24E-07 |
| COL1A2 | -4.90206 | -29.8998 | 2.54E-11 |
| COL3A1 | -4.91636 | -30.1975 | 7.88E-12 |
| CPZ | -5.02628 | -32.5882 | 2.26E-07 |
| SCD | -5.13401 | -35.1149 | 0 |
| C1QTNF6 | -5.13938 | -35.2459 | 0 |
| COL1A1 | -6.18835 | -72.9253 | 0 |
